# Arthrospira Platensis Attenuates Endothelial Inflammation and Monocyte Activation

**DOI:** 10.3390/ijms26167844

**Published:** 2025-08-14

**Authors:** Ilaria Leone, Valentino Costabile, Giovanni Smaldone, Monica Franzese, Andrea Soricelli, Anna D’Agostino

**Affiliations:** 1 IRCCS SYNLAB SND, 80143 Naples, Italy; ilaria.leone@synlab.it (I.L.); valentino.costabile@synlab.it (V.C.);; 2 Department of Medical, Movement and Well-Being Sciences, University of Naples Parthenope, 80133 Naples, Italy

**Keywords:** spirulina platensis, atherosclerosis, endothelial inflammation, monocyte adhesion, NF-κB signaling, VEGF pathway

## Abstract

Cardiovascular diseases (CVDs) remain the leading cause of morbidity and mortality in industrialized countries. Coronary artery disease (CAD) represents the most prevalent form of cardiovascular disease and remains a leading cause of morbidity, mortality, and long-term disability worldwide. Therefore, the identification of early biomarkers and clarification of the mechanism of action of pharmacological adjuvants is urgently needed. Nutraceuticals such as Arthrospira platensis (commonly known as spirulina) have emerged as promising modulators for their notable vascular anti-inflammatory properties. In this study, we provide novel evidence of the anti-inflammatory and anti-atherosclerotic potential of Arthrospira platensis toward endothelial cells and immune interactions, combining in vitro assays with bioinformatic profiling. Spirulina treatment significantly attenuated endothelial and angiogenic activation, reducing pro-inflammatory cytokine and VEGFA/VEGFR2 expression. Additionally, it also decreased the activation and adhesion capabilities of THP-1 monocytic cell lines. Through computational analysis of the complex molecular mixture present in Arthrospira platensis, we have identified a subset of compounds exhibiting high structural similarity to CHEMBL3559503, a well-characterized synthetic molecule with dual activity as a TLR9 agonist and anti-angiogenic agent. This represents a novel insight, suggesting that spirulina may serve as a natural source of analogues capable of modulating both immune and angiogenic pathways. These results highlight Arthrospira platensis as a promising candidate nutraceutical for targeting endothelial/immune crosstalk in the context of atherosclerosis prevention, offering both mechanistic insights and translational potential.

## 1. Introduction

CVDs are the leading cause of morbidity and mortality in industrialized countries. CADs are the most common type of cardiovascular disease and are characterized by the narrowing or blockage of coronary arteries due to the progressive accumulation of atherosclerotic plaques. Endothelial dysfunction, lipid buildup, persistent inflammation, and smooth muscle cell proliferation are the main causes of this degenerative process, which eventually results in decreased myocardial perfusion and an elevated risk of acute coronary events including myocardial infarction and sudden cardiac death. According to the World Health Organization, CAD remains the leading cause of death globally, responsible for an estimated 9 million deaths in 2019 alone [1,2,3]. Modifiable lifestyle behaviours, such as smoking, physical inactivity, and unhealthy dietary habits, are known contributors to atherosclerosis development [4]. These factors promote chronic systemic inflammation and metabolic imbalance, which are key drivers of endothelial dysfunction and plaque formation [5].

The earliest step in this pathological cascade is endothelial activation, which facilitates the adhesion and transmigration of monocytes into the subendothelial space, where they differentiate into macrophages and uptake oxidized low-density lipoproteins (oxLDLs), forming lipid-laden foam cells [6,7]. These foam cells not only contribute to plaque expansion but also release pro-inflammatory cytokines and matrix-degrading enzymes, promoting plaque instability and increasing the risk of rupture and thrombosis [8].

To address the global burden of CVDs, there is an urgent need to better understand the underlying molecular mechanisms of atherosclerosis and to identify therapeutic targets [9,10].

Vascular endothelial growth factor A (VEGFA) and its main receptor VEGFR2 are key mediators in atherosclerosis, as they regulate endothelial responses to inflammation and damage [11,12]. Inflammatory stimuli such as TNF-α and IL-1β promote the expression of VEGFA in endothelial cells, contributing to pathological angiogenesis and increased vascular permeability, features commonly observed in unstable atherosclerotic plaques [13]. Although VEGFA/VEGFR2 signalling plays physiological roles in endothelial survival and regeneration, its overactivation under chronic inflammatory conditions can promote leukocyte recruitment, neovascularization, and intraplaque hemorrhage [14,15], contributing to plaque instability. In this context, targeting VEGFA/VEGFR2 pathways may help preserve vascular integrity and reduce disease progression. These signalling events involve downstream activation of PI3K/Akt and MAPK cascades, which modulate endothelial cell proliferation, migration, and oxidative stress response. However, when dysregulated, they may fuel chronic vascular inflammation and maladaptive angiogenesis.

Dietary interventions rich in bioactive natural compounds have gained attention as complementary strategies to reduce cardiovascular risk [16]. Numerous nutraceuticals have shown effectiveness in modulating major risk factors, including blood pressure, lipid profiles, oxidative stress, and inflammation [2,17,18,19,20,21]. For instance, anthocyanin-rich fruits and omega-3 polyunsaturated fatty acids from fatty fish have demonstrated endothelial protective effects through antioxidant and anti-inflammatory mechanisms [21,22]. Among promising nutraceuticals, Arthrospira platensis (commonly known as spirulina), a Cyanobacterium rich in phycocyanin, essential amino acids, and antioxidants, has shown potential cardiovascular benefits [23,24]. Previous studies suggest its ability to modulate endothelial activation markers such as VCAM-1 and E-selectin, yet its broader effects on vascular inflammation and immune cell recruitment remain underexplored [18].

Based on these insights, our study aimed to elucidate the mechanisms by which spirulina may influence endothelial and monocyte activation, integrating in vitro and computational approaches.

## 2. Results

### 2.1. Spirulina Treatment Does Not Induce Cytotoxicity in HUVECs

**Figure 1:**
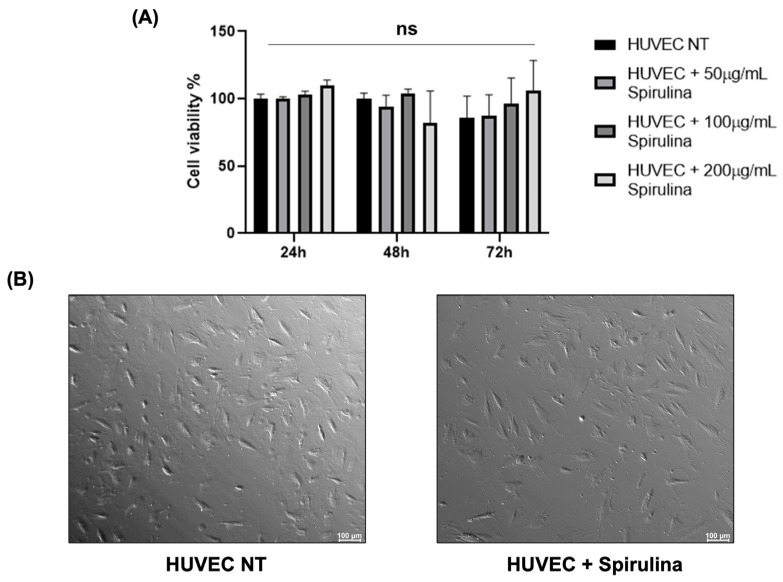
Cytotoxic effects of spirulina treatments on HUVECs. (**A**) Cell viability rate of HUVECs by MTS assay to evaluate spirulina cytotoxic effect. Three replicates of each sample were analyzed by three independent experiments. Data are presented as mean bars ± SEM. The *p*-values were calculated using a 2-way ANOVA test (ns, not significant); (**B**) bright-field microscopy image of HUVEC NT and HUVECs after 72 h pretreatment with 50 µg/mL spirulina. Images were acquired using the Leica MICA Microhub system with a 10× magnification.

To assess the potential cytotoxic effects of spirulina on HUVECs, we dissolved spirulina powder in PBS (10 mg/mL) and added it to the culture medium at final concentrations of 50, 100, and 200 µg/mL. MTS viability assays were performed at 24, 48, and 72 h post-treatment. As shown in Figure 1A, no statistically significant cytotoxicity was observed at any time point. Moreover, HUVECs imaged under bright-field microscopy at 72 h showed no appreciable morphological alterations following spirulina treatment compared to untreated controls (Figure 1B).

### 2.2. Spirulina Attenuates Endothelial Inflammation and Modulates Angiogenic Signalling in HUVECs

**Figure 2:**
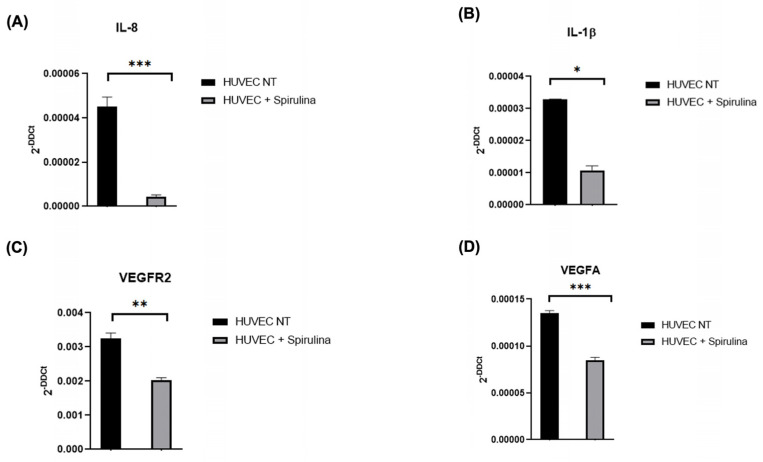
qRT–PCR analysis of the mRNA levels. Changes in the relative expression in the HUVEC line after 72 h pretreatment with 50 µg/mL spirulina of (**A**,**B**) pro-inflammatory cytokines (IL-8 and IL-1β) and (**C**,**D**) vascular biomarkers (VEGFR2 and VEGFA). The values are mean ± SEM of three independent experiments, normalized by the expression of GAPDH. The *p*-value was calculated by a *t*-test with 0.001 ≤ *p ≤* 0.1. (* *p* < 0.05, ** *p* < 0.01, *** *p* < 0.001).

To explore the anti-inflammatory and vascular modulatory potential of spirulina in HUVECs, we measured the expression of selected biomarkers following 72 h treatment with 50 µg/mL spirulina. Expression levels of IL-8 and IL-1β were significantly reduced compared to untreated controls (Figure 2A,B). Additionally, VEGFA and VEGFR2, key players in angiogenic signalling, were also downregulated in treated cells (Figure 2C,D), indicating a dual anti-inflammatory and anti-angiogenic effect of spirulina.

### 2.3. Spirulina Attenuates Monocyte Activation and Foam Cell Formation in THP-1 Cells

**Figure 3:**
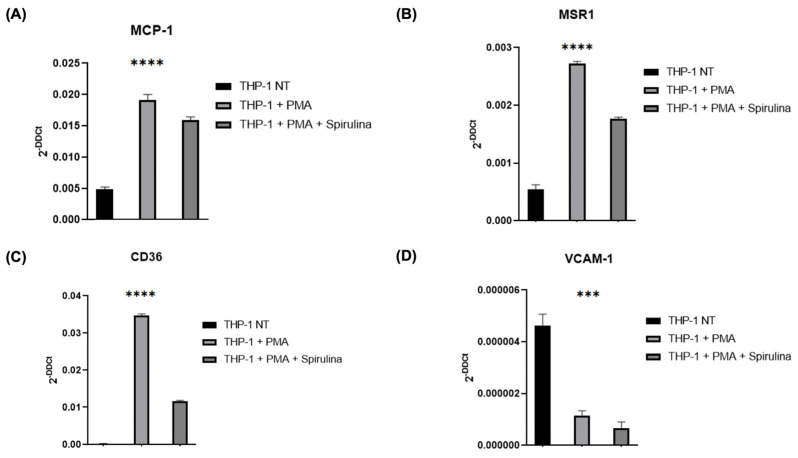
qRT–PCR analysis of the mRNA levels of THP-1 cells. Changes in the relative expression in the THP-1 cell line after PMA and spirulina treatment of (**A**) chemokines (MCP-1); (**B**,**C**) scavenger receptors (MSR1, CD36); and (**D**) biomarker of vascular cell adhesion (VCAM-1). The values are mean  ±  SEM of three independent experiments, normalized by the expression of GAPDH. The *p*-values were calculated using a one-way ANOVA test (*** *p* < 0.001; **** *p* < 0.0001).

Given the role of monocytes in plaque formation, we investigated spirulina’s effects on THP-1 differentiation and foam cell-associated markers. After confirming the absence of cytotoxicity at 100 µg/mL (Figure S1), THP-1 cells were differentiated into macrophages with PMA in the presence or absence of spirulina. Treatment resulted in reduced expression of MCP-1 (Figure 3A), as well as the scavenger receptors MSR1 and CD36, both key to oxLDL uptake (Figure 3B,C). Notably, VCAM-1 expression was also significantly decreased in spirulina-treated cells (Figure 3D), suggesting reduced activation and adhesion potential of these macrophages.

### 2.4. Spirulina Attenuates NF-κB Signalling in HUVECs Under Basal and TNF-α-Induced Conditions

**Figure 4:**
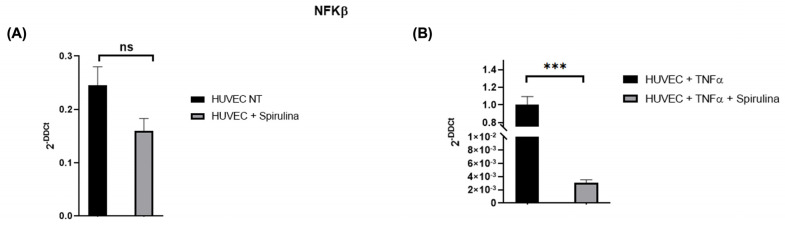
qRT–PCR analysis of the mRNA levels of NFKβ. Changes in the relative expression of NFKβ in the HUVEC line after spirulina treatment with (**A**) and without (**B**) TNFα. The values are mean ± SEM of three independent experiments, normalized by the expression of GAPDH. The *p*-value was calculated by a *t*-test with 0.001 ≤  *p* ≤ 0.1 (ns = not significant; *** *p* < 0.001).

To assess NF-κB involvement, we measured its expression in HUVECs treated with spirulina (50 µg/mL, 72 h). Spirulina treatment led to a reduction in NF-κB expression under both basal (Figure 4A) and TNF-α-stimulated conditions (Figure 4B), suggesting that spirulina may attenuate pro-inflammatory NF-κB signalling in endothelial cells.

### 2.5. Spirulina Attenuates Monocyte Adhesion to Activated Endothelium

**Figure 5:**
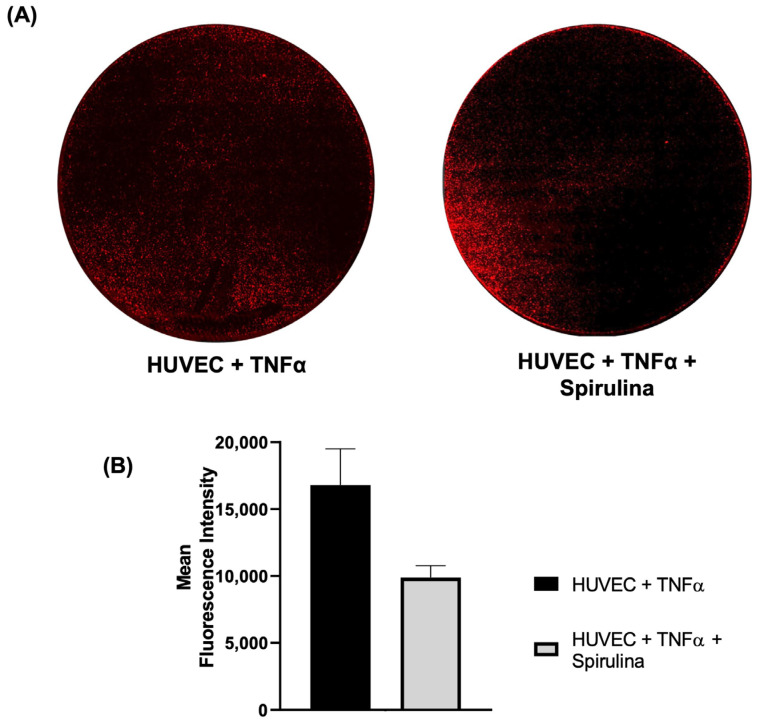
Monocyte–endothelial adhesion assay. (**A**) Confocal images of fluorescence of monocytes labelled with HLA (red) and (**B**) the mean of fluorescence intensity. Images were acquired using the Leica MICA Microhub system with 10× magnification (mosaic view).

We next evaluated whether spirulina affects monocyte adhesion to endothelial cells under inflammatory conditions. HUVECs were pre-treated with spirulina and stimulated with TNF-α, followed by a monocyte adhesion assay. The number of adhered monocytes was significantly reduced in the spirulina-treated condition compared to TNF-α-only controls (Figure 5A,B), indicating a potential role for spirulina in reducing endothelial–leukocyte interactions during early atherogenesis. 

### 2.6. Structural Similarity Analysis Identifies Functional Mimicry Between Spirulina Metabolites and HUVEC-Interacting Molecules

**Figure 6:**
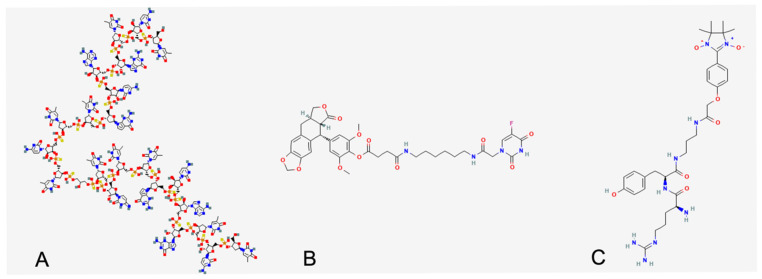
Report HUVEC effector with the highest number of structurally related compounds from spirulina extract. (**A**) CHEMBL355950 (158 similar small molecules) has been annotated as capable of inhibiting VEGF-stimulated network formation in HUVEC and thus interfering with neo-angiogenesis and chemotaxis; (**B**) CHEMBL3780449 (35 similar small molecules) can downregulate MMP-2 expression in HUVEC and thus preserve extracellular matrix, which indeed interferes with leukocyte adhesion; (**C**) CHEMBL3787168 (33 similar small molecules) recognized as capable of attenuating tissue damage induced by ROS during ischemia/reperfusion events.

To explore molecular mimicry between spirulina metabolites and known HUVEC regulators, we applied a computational analysis pipeline. Starting with 690 proteins from *A. platensis* (UniProt ID: 118562), we retrieved 508 unique Rhea reaction identifiers. From ChEMBL, 1845 active HUVEC-related assays corresponding to 842 chemical compounds were extracted after filtering for functional relevance. Structural similarity was quantified using MACCS molecular fingerprints and DICE coefficients. This led to the identification of 593 matches between 199 spirulina compounds and 89 HUVEC modulators (Figure 6 and Supplementary Tables S1 and S2). The top 20 HUVEC regulators with the highest number of matching spirulina metabolites are listed in Table 1. Among these, CHEMBL3559503 (Table 2) showed notable similarity with 15 distinct spirulina metabolites, suggesting shared functional properties.

## 3. Discussion

Chronic inflammation plays a pivotal role in the pathogenesis of numerous diseases, notably cancer and atherosclerosis, two of the most prevalent causes of morbidity and mortality in industrialized countries [25,26]. Atherosclerosis, in particular, is now widely recognized as a chronic inflammatory condition of the arterial wall and remains a leading driver of CVDs worldwide [27]. In this context, the identification of natural compounds with low toxicity profiles and immunomodulatory activity represents a valuable strategy for the development of adjunctive therapies aimed at mitigating CVD progression and improving patient outcomes [2,19,21].

In our study, we provide experimental evidence that spirulina extracts a modulatory effect on both endothelial and monocyte/macrophage response, key cellular players in atherogenesis. Specifically, spirulina treatment led to a significant downregulation of maturation markers in THP-1-derived macrophages [28], along with reduced expression of scavenger receptors involved in oxLDL internalization, such as MSR1 and CD36 [29]. These effects suggest the capacity of spirulina to interfere with foam cell formation and monocyte activation, thereby attenuating early pro-atherogenic processes [30].

In parallel, HUVECs exposed to spirulina displayed lower expression levels of pro-inflammatory cytokines such as IL-8 and IL-1β, as well as reduced expression of angiogenic mediators like VEGFA and VEGFR2 [31,32]. These results point to a dual anti-inflammatory and anti-angiogenic activity of spirulina, which may contribute to vascular homeostasis and plaque stabilization in inflammatory settings.

Our data demonstrate a clear downregulation of pro-inflammatory cytokines such as IL-1β and IL-8 in endothelial cells treated with spirulina, and it is important to note that we did not observe statistically significant changes in the expression levels of other key inflammatory markers, such as IL-6 and IL-10. The absence of modulation in these cytokines suggests that the anti-inflammatory effects of spirulina may not be broad-spectrum but instead selectively targeted toward specific pathways or mediators. IL-8 and IL-1β are closely associated with acute endothelial activation and leukocyte recruitment, which is highly relevant in early atherogenic events. The selective inhibition of these markers may therefore reflect a more focused immunomodulatory mechanism.

Although the current study focused primarily on the modulation of VEGFA/VEGFR2 expression, VEGFR2 signalling is known to regulate key downstream pathways such as PI3K/Akt and MAPK, which mediate endothelial proliferation, migration, and survival. While our in vitro experiments did not directly assess these signalling cascades, emerging evidence from the literature suggests that spirulina-derived bioactive compounds, including phycocyanin and peptides, may influence these pathways [33]. For instance, phycocyanin has been shown to inhibit PI3K/Akt signalling in tumour models, while other protein components of spirulina have been associated with MAPK activation in epithelial cells [34]. These data open up new hypotheses on the potential intracellular targets of spirulina in vascular contexts. Further studies involving phospho-protein profiling or pathway-specific inhibitors are necessary to dissect whether the observed anti-inflammatory and anti-angiogenic effects are mediated through these classical VEGFR2 downstream effectors.

To further explore the molecular basis of spirulina’s biological activity, we performed a computational analysis comparing spirulina-derived metabolites with known endothelial modulators. Among the identified candidates, a cluster of 158 spirulina metabolites showed high structural similarity to CHEMBL3559503, a synthetic immunomodulatory oligonucleotide (IMO) with established anti-angiogenic effects via VEGF/VEGFR2 pathway inhibition [35]. Given that CHEMBL3559503 is known to suppress endothelial proliferation and neovascularization, the structural resemblance suggests that certain spirulina metabolites might act on similar signalling cascades, thus contributing to the observed changes in vitro.

To our knowledge, this is the first study to report a structural convergence between spirulina-derived natural metabolites and a synthetic anti-angiogenic compound (CHEMBL3559503), which targets the VEGF–VEGFR2 pathway. This novel insight provides a molecular rationale for the observed in vitro effects and suggests that spirulina may harbour compounds with therapeutic potential similar to known synthetic angiogenesis inhibitors.

Taken together, our findings support a mechanistic framework in which spirulina exerts pleiotropic vascular-protective effects, potentially through the inhibition of inflammatory signalling and angiogenic activation. The structural mimicry between spirulina metabolites and known endothelial inhibitors provides a molecular rationale for the bioactivities observed in HUVECs and macrophages. These insights highlight the therapeutic potential of spirulina not only as a dietary supplement but also as a promising reservoir of bioactive molecules for future drug development targeting vascular inflammation and atherosclerosis.

## 4. Materials and Methods

### 4.1. Cell Lines and Culture Conditions

Primary Human Umbilical Vein Endothelial Cells HUVECs (PCS-100-010) and THP-1 (TIB-202) were provided by ATCC. HUVECs were grown in an Endothelial Cell Medium kit (Chicago, IL, USA, Cat. #M1168) supplemented with VEGF, ECGS, Heparin, EGF, hydrocortisone, antibiotic–antimycotic solution, and fetal bovine serum. All components are included in the kit. THP-1 cells were cultured according to the manufacturer’s recommendations. Briefly, the cells were grown in RPMI 1640 (GIBCO, Billings, MT, USA, #11875093) supplemented with 10% FBS, 100 U/mL penicillin, 100 mg/mL streptomycin, and 1% L-glutamine. Cells were grown at 37 °C in a 5% CO_2_ atmosphere.

### 4.2. Preparation of Spirulina Extract

We obtained 100% natural caps of Arthrospira platensis from BioSpira Srl (BioSpira, Alvignano, Italy, caps Lot code 231211). The powder was dissolved in PBS to a final concentration of 10 mg/mL and appropriately diluted at the time of the experiments. The solution was then centrifuged at 3400× *g* for 5 min, followed by filtration through 0.22 µm filters. The extracts were stored at 4 °C.

### 4.3. MTS Assay

The [3-(4,5-dimethylthiazol-2-yl)-5-(3-carboxymethoxyphenyl)-2-(4-sulfophenyl)-2H tetrazolium] assay (CellTiter 96 Aqueous One Solution Cell Proliferation Assay, Promega, Madison, WI, USA, Cat. # G3580) was used to assess cytotoxicity according to the manufacturer’s protocol. The absorbance was detected at 490 nm by a Multilabel Reader luminescence (Victor Nivo Multimode Microplate Reader, PerkinElmer, Waltham, MA, USA).

### 4.4. Monocyte–Endothelium Adhesion Assay

For the monocyte–endothelial assay, HUVEC and THP-1 cells were cultured according to manufacturer guidelines. HUVEC populations were plated in 24 wells at a density of 3 × 10^4^ and cultured with or without spirulina 50 μg/mL for 72 h. After 72 h, the culture medium was removed, cells were washed, and fresh medium supplemented with TNF-alpha (10 ng/mL) was added to induce a pro-inflammatory stimulus. After 4 h of TNF-alpha exposure, the cells were washed again and incubated with fresh medium. Subsequently, 3 × 10^6^ THP-1 cells, pre-labelled with a fluorescent anti-HLA antibody (1:50 in 1% PBS; Beckman Coulter, Brea, CA, USA, HLA-DR-PC5, #A 07793), were added to the activated endothelium. The THP-1 cells were allowed to adhere to the inflamed endothelium for 1 h at 37 °C. After the incubation period, non-adherent cells were removed, and plates were washed to completely remove non adherent THP-1. Cells were fixed with 4% paraformaldehyde (PFA), and images were acquired using the MICA confocal microhub microscope at 10× magnification (Leica Microsystems, Wetzlar, Germany).

### 4.5. RNA Extraction and Real-Time PCR

Cells were collected in TRIzol Reagent (Invitrogen, Waltham, MA, USA, #15596026). Total RNA was extracted according to the manufacturer’s protocol, then 500 ng of total RNA was used to synthesize cDNA using the Retro SensiFAST cDNA Synthesis Kit (Meridian Biosciences, Cincinnati, OH, USA, #BIO 65054), following the manufacturer’s instructions. Real-time PCR analysis was performed using iQ SYBR Green Supermix (Qiagen, Hilden, Germany, #1708882) in a CFX96 Real-Time PCR Detection System (BIORAD, Hercules, CA, USA). For each gene, values are presented as mean ± SD of three independent experiments, and gene expression was evaluated by normalizing the Ct values of target genes on the GAPDH reference gene. To calculate the relative expression levels, the 2-DDCT method was used. Statistical analyses were performed using GraphPad Prism Version 9. The values are mean ± SEM of three independent experiments, normalized by the expression of GAPDH. The *p*-value was calculated by the pairwise *t*-test 0.001 ≤ *p* ≤ 0.1 or ANOVA for multiple comparisons (* *p* < 0.05, ** *p* < 0.01, *** *p* < 0.001).

### 4.6. Bioinformatics Analysis

In silico bioinformatics analysis was conducted to identify biochemical agents potentially involved in observed cellular behaviours. This approach leverages the principle of ligand mimicry, where molecules with similar chemical structures can interact with the same biological receptor. By comparing chemical structures, we aimed to identify molecules with overlapping properties and functions. Specifically, we focused on comparing spirulina metabolites with molecules known to affect Human Umbilical Vein Endothelial Cells (HUVECs) to pinpoint spirulina extract components with a potential role in HUVEC modulation.

Spirulina protein content was retrieved from the UniProt database (Available online: https://www.uniprot.org/ (accessed on 13 June 2025), taxonomy ID 118562). This dataset was enriched with metadata from Rhea DB (Available online: https://www.rhea-db.org/ (accessed on 13 June 2025)), which provides information on chemical compounds participating in reactions associated with these proteins (Supplementary Table S1). Reaction identifiers from Rhea DB, combined with its metadata files, were used to parse and retrieve the SMILES (Simplified Molecular Input Line Entry System) representation of chemical compounds involved, which was necessary for subsequent biochemical structure comparisons. Concurrently, biomolecules known to interact with HUVECs were retrieved from the ChEMBL database (available online: https://www.ebi.ac.uk/chembl/ (accessed on 13 June 2025)) using their cell-line-specific ChEMBL ID (CHEMBL613979).

### 4.7. Biochemical Structural Comparison

All collected molecules were managed using custom Python scripts V 3.13.0. The chembl_web_client package facilitated ID cross-mapping (e.g., ChEMBL to ChEBI), while the RDKit package was used for digital molecule representation, molecular fingerprint generation, and structural similarity comparison. Molecule SMILES were canonicalized to improve ID retrieval and fingerprint generation. Molecular fingerprints were computed using the MACCS method, and their relative similarity was assessed by calculating their DICE coefficient (both steps were performed using RDKit). Spirulina compounds showing a structural similarity with HUVEC interactors higher than 80% were stored and grouped under the ChEMBL ID associated with the HUVEC interactors. The ChEMBL ID groups obtained were sorted by their size, and the top 20 groups were retrieved and annotated with their experimental abstracts (Table 1).

## 5. Conclusions and Future Directions

This study highlights a previously uncharacterized structural convergence between a synthetic anti-angiogenic compound (CHEMBL3559503), known to act on the VEGF–VEGFR2 signalling pathway, and several naturally occurring metabolites derived from Spirulina platensis. This structural similarity may provide a mechanistic rational for the anti-inflammatory and anti-angiogenic effects observed in vitro. Building on these findings, future work will aim to isolate and purify the spirulina-derived metabolites showing the highest structural similarity to CHEMBL3559503. These purified compounds will be further investigated for their potential to modulate the VEGFA–VEGFR2 axis and suppress macrophage maturation and pro-inflammatory activation.

Despite these encouraging results, several limitations must be acknowledged. First, all experiments were performed in vitro using established cell lines. While these models are useful for initial mechanistic insights, they do not fully replicate the complexity of the in vivo environment, which involves systemic immune responses, metabolic factors, and tissue-specific interactions. Second, we used a single endothelial cell type (HUVECs) and a monocytic cell line (THP-1), which may not fully represent the heterogeneity and physiological behaviour of primary human cells.

To overcome these limitations, future research should include in vivo models of atherosclerosis and inflammation to evaluate the systemic effects, bioavailability, and safety profile of spirulina-derived compounds. Moreover, clinical studies involving patients at elevated cardiovascular risk are needed to evaluate the efficacy of spirulina-based nutraceutical interventions in real-world settings. Stratification of participants based on inflammatory status or genetic risk factors may also help identify subgroups most likely to benefit from such interventions, contributing to more targeted and personalized approaches to cardiovascular disease prevention.

## Figures and Tables

**Table 1:** Ranked groups of spirulina metabolite resembling known HUVEs interactors.

Combined	Assay Description	Combined Count
CHEMBL3559503	Inhibition of VEGF-stimulated network formation in HUVEC at 1 uM	158
CHEMBL3780449	Downregulation of MMP-2 expression in HUVECs at 0.2 to 0.5 uM after 24 h by Western blot analysis	35
CHEMBL3787168	Inhibition of simulated ischemia/reperfusion-induced human HUVEC death assessed as increase in tubular mitochondria levels at 30 ug/mL after 2 h by Hoechst 33, 342 dye-based fluorescence assay (Rvb = 11.3 ± 2.1%)	33
CHEMBL3786354	Inhibition of simulated ischemia/reperfusion-induced cytochrome C release in HUVECs at 30 ug/mL after 2 h by fluorescence assay (Rvb = 75.3 ± 5.2%)	25
CHEMBL4845725	Pro-angiogenic activity in HUVECs assessed as organization of capillary network formation by measuring increase in branch point number count at 50 nM measured after 6 h by inverted microscopy relative to Ac-HPLW-NH2	20
CHEMBL1630373	Antiangiogenic activity in HUVECs cocultured with human PaSMC assessed as reduction in total tube length at 125 nM after 72 h	19
CHEMBL3115403	Induction of proapoptosis in VEGF-treated HUVECs assessed as increase in caspase 3 activity at 25 to 100 ng/mL after 8 h by fluorimetric assay	19
CHEMBL4063339	Protection against simulated ischemia/reperfusion injury-induced cell death in HUVECs assessed as increase in cell viability at 30 ug/mL treated for 12 h under normoxic condition prior to simulated ischemia for 2 h followed by compound washout measured 3 h post reperfusion	13
CHEMBL4070823	Protection against simulated ischemia/reperfusion injury-induced cell death in HUVECs assessed as increase in cell viability at 30 ug/mL treated for 12 h under normoxic condition prior to simulated ischemia for 2 h followed by compound washout measured 3 h post reperfusion	13
CHEMBL4780091	Cytotoxicity against HUVEC assessed as cell viability at 100 uM after 72 h by MTT assay relative to control	13
CHEMBL4074157	Protection against simulated ischemia/reperfusion injury-induced cell death in HUVECs assessed as increase in cell viability at 30 ug/mL treated for 12 h under normoxic condition prior to simulated ischemia for 2 h followed by compound washout measured 3 h post reperfusion	12
CHEMBL4073555	Protection against simulated ischemia and reperfusion injury-induced mitochondrial oxidative damage in HUVECs assessed as reduction in cytochrome c release at 30 ug/mL treated for 12 h under normoxic condition prior to simulated ischemia for 2 h followed by compound washout measured 3 h post reperfusion by MitoProbe-based immuno-fluorescence assay (Rvb = 71.2 ± 4.5%)	11
CHEMBL4075508	Protection against simulated ischemia/reperfusion injury-induced mitochondrial oxidative damage in HUVECs assessed as increase in tubular mitochondrial cells at 30 ug/mL pretreated for 12 h followed by compound washout and subsequent ischemia simulation for 2 h in presence of compound prior to 3 h reperfusion under drug-free medium by MitoProbe-based confocal microscopic analysis	11
CHEMBL4076966	Protection against simulated ischemia/reperfusion injury-induced cell death in HUVECs assessed as increase in cell viability at 30 ug/mL treated for 12 h under normoxic condition prior to simulated ischemia for 2 h followed by compound washout measured 3 h post reperfusion	11
CHEMBL412927	Downregulation of VEGFR2 expression in VEGFA-stimulated HUVECs at 5 to 20 uM after 24 h by Western blot analysis	9
CHEMBL1083318	Antiinvasive activity in VEGF-stimulated HUVECs assessed as cellular migration after 24 h by fluorescence assay relative to control	7
CHEMBL4238261	Antimigratory activity in HUVECs at 25 uM after 8 h by crystal violet staining-based Boyden chamber assay relative to control	7
CHEMBL4860306	Anti-angiogenic activity against HUVECs assessed as inhibition of tubular structure formation at 10 uM incubated for 6 days by Matrigel-based phase-contrast microscopic analysis	7
CHEMBL1085966	Antiinvasive activity in VEGF-stimulated HUVECs assessed as cellular migration after 24 h by fluorescence assay relative to control	5
CHEMBL1085981	Antiinvasive activity in VEGF-stimulated HUVECs assessed as cellular migration after 24 h by fluorescence assay relative to control	5

Each group corresponds to a CHEMBL-annotated compound known to interact with endothelial (HUVEC) targets. Group size indicates the number of spirulina metabolites with significant structural similarity to the corresponding CHEMBL entity. Each entry is annotated based on its primary bioassay or known target interaction.

**Table 2:** Top 15 spirulina molecules similar to CHEMBL3559503.

HUVEC_ChemblID	Spirulina_ChebiID	Similarity
CHEMBL3559503	CHEBI: 63224	0.90797546
CHEMBL3559503	CHEBI: 77896	0.90797546
CHEMBL3559503	CHEBI: 191199	0.902439024
CHEMBL3559503	CHEBI: 57692	0.896969697
CHEMBL3559503	CHEBI: 58897	0.896551724
CHEMBL3559503	CHEBI: 57364	0.885057471
CHEMBL3559503	CHEBI: 62727	0.879518072
CHEMBL3559503	CHEBI: 78513	0.879518072
CHEMBL3559503	CHEBI: 57527	0.87654321
CHEMBL3559503	CHEBI: 57964	0.875
CHEMBL3559503	CHEBI: 57288	0.873563218
CHEMBL3559503	CHEBI: 58342	0.872093023
CHEMBL3559503	CHEBI: 57384	0.872093023
CHEMBL3559503	CHEBI: 57287	0.870588235
CHEMBL3559503	CHEBI: 57328	0.870588235

## Data Availability

Part of the data presented in this study were obtained from [ChEMBL] at [https://www.ebi.ac.uk/chembl/] [accessed on 13 June 2025]. All other original data are available from the corresponding author upon reasonable request.

## Supplementary Materials

The following supporting information can be downloaded at https://www.mdpi.com/article/10.3390/ijms26167844/s1.
