# Supplementary figures and images for: Arthrospira Platensis Attenuates Endothelial Inflammation and Monocyte Activation
Source: Int J Mol Sci. 2025 Aug 14;26(16):7844. doi: 10.3390/ijms26167844 (PMC12386423; doi:10.3390/ijms26167844)

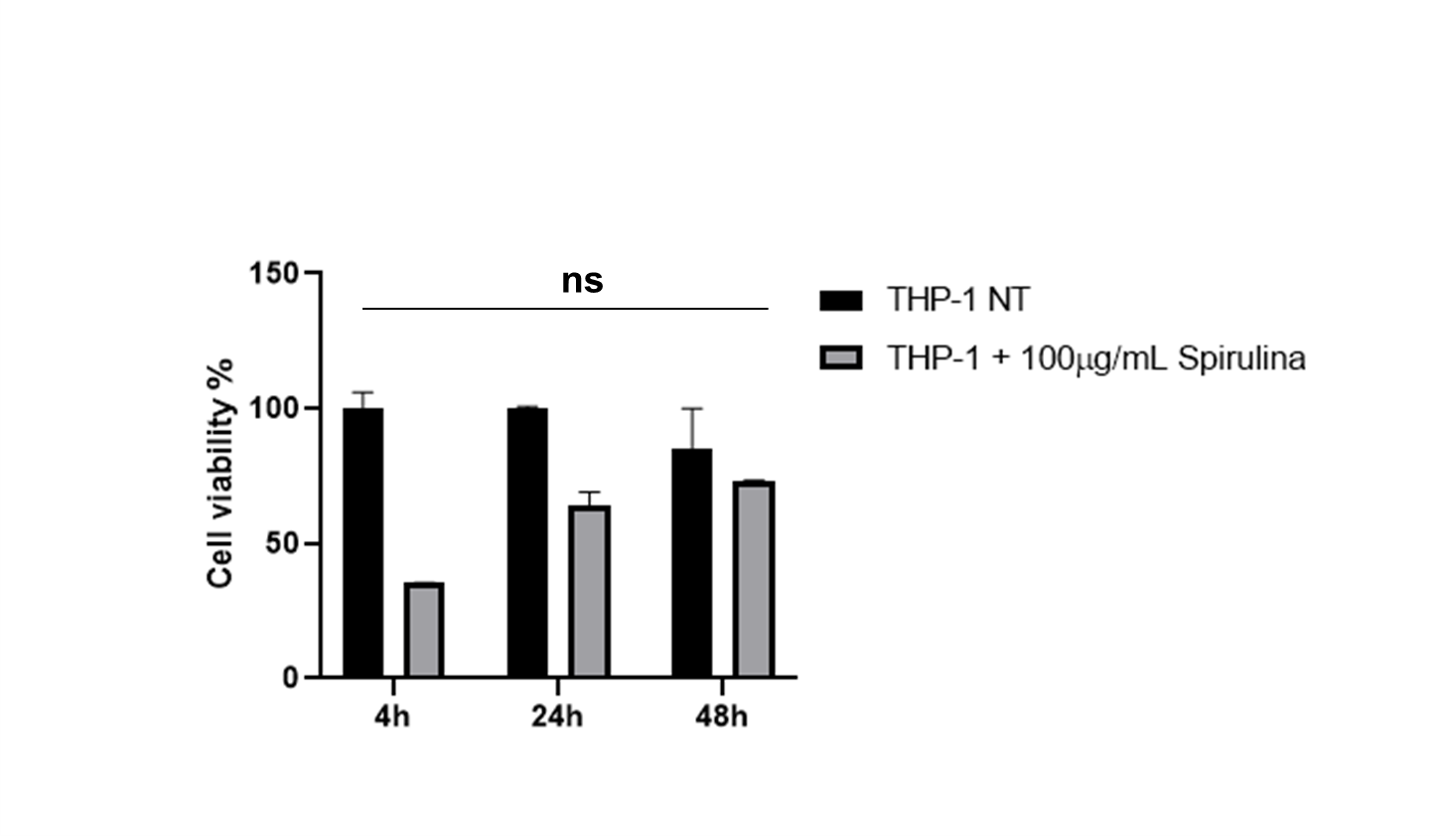

Supplement: Supplementary file 1 [file ijms-26-07844-s001.zip › ijms-3769013 Figure S1.tiff]
